# Effect of Persulfate Activation by Electrogenerated H_2_O_2_ and Anodic Oxidation on the Color Removal of Dye Solutions at Pt and BDD Anodes

**DOI:** 10.3390/ijerph192315688

**Published:** 2022-11-25

**Authors:** Yifan Yao, Kai Zhu, Yucan Liu, Qianjin Liu, Lihua Huang

**Affiliations:** 1 Shandong Provincial Key Laboratory of Water and Soil Conservation and Environmental Protection, College of Resources and Environment, Linyi University, Linyi 276000, China; 2 School of Civil Engineering, Yantai University, Yantai 264005, China

**Keywords:** anodic oxidation, electrogenerated H_2_O_2_, persulfate, hydroxyl radical, sulfate radical

## Abstract

In this study, tartrazine solutions were oxidized using innovative electrochemical advanced oxidation processes (EAOPs) that combined persulfate (PS) activation with electrogenerated H_2_O_2_, cathodic reduction and anodic oxidation at Pt and BDD anodes, and graphite cathode in an undivided stirred reactor. For the Pt anode, SO_4_**·**^−^ was generated from a reduction reaction at the cathode and a reaction between the PS and electrogenerated H_2_O_2_. For the BDD anode, SO_4_**·**^−^ was generated from a reduction reaction at the cathode, an oxidation reaction at the anode, and a reaction between PS and electrogenerated H_2_O_2_. Among these activation methods, the activation efficiency of PS by electrogenerated H_2_O_2_ is much better than other methods. The effects of PS concentration up to 36 mM, applied current density between 6 to 15 mA cm^−2,^ and temperatures between 25 to 45 °C were investigated. For the electro-Fenton process with Pt anode (Pt-H_2_O_2_/PS process), the best result for oxidizing 250 mg L^−1^ tartrazine solution was obtained with 37.5 mM Na_2_SO_4_ + 9.0 mM Na_2_S_2_O_8_, applied current density at 12 mA cm^−2^ and 45 °C, acquiring total color removal after 30 min reaction. For the electro-Fenton process with BDD anode (BDD-H_2_O_2_/PS process), the best result for oxidizing 250 mg L^−1^ tartrazine solution was obtained with 25 mM Na_2_SO_4_ + 18 mM Na_2_S_2_O_8_, applied current density at 12 mA cm^−2^ and 45 °C, yielding 100% color removal after 30 min reaction. The main oxidizing agents are SO_4_**·**^−^ and OH**·** in the anodic oxidation process with PS and the electro-Fenton process with PS. It is concluded that the additions of PS tremendously improve the oxidation power of electro-Fenton processes with PS, especially the Pt-H_2_O_2_/PS process.

## 1. Introduction

Textile manufacturing industries produce large amounts of wastewater containing high concentrations (up to 250 mg L^−1^) of soluble azo dyes [1]. These high-concentration contaminations cannot be significantly removed by conventional wastewater treatment plants owing to their stable chemical structure’s resistance to biodegradation [2]. As a result, many types of azo dyes have been detected in reservoirs, rivers, and even drinking water [3]. Although the carcinogenic, mutagenic, and teratogenic effects of most azo dyes remain unknown, heightened concerns still exist concerning the potential adverse consequences on living organisms, including human beings [4].

Recently, electrochemical advanced oxidation processes (EAOPs) have attracted increasing interest as promising technologies for the degradation of azo dyes, providing several characteristic advantages, such as easy operation, simple equipment, and ambient temperature and pressure [5,6]. As with the most popular and convenient EAOPs, anodic oxidation (AO) is used to degrade contaminations by directly oxidation through electron transfer and/or indirectly oxidation through electrogenerated reactive species (hydroxyl radical) from simultaneous oxidation of water molecules at the anode surface A, as described in Equation (1) [7,8]. In reality, indirect oxidation mechanisms involving water intermediates are the convincing paradigm for the AO process at anodes based on recently developed superior boron-doped diamond (BDD) [9]. However, at an anode based on noble metal (Pt), A-OH**·** can be further oxidized to higher oxide (A=O). Hence, at Pt anode, degradation is a mediated electrolysis with organics (R) oxidized by A=O, regenerating the reduced anode surface A, as described in Equations (2) and (3) [10]. During the AO process, the degradation efficiencies of azo dyes rely largely on the properties of the anode material [11]. However, the effect of the cathode is usually neglected, assuming it is the only counter electrode for the reduction reaction from H_2_O to H_2_. This situation is thoroughly changed when a carbonaceous cathode fed with O_2_ is employed during the AO process since H_2_O_2_ is generated from O_2_ through a 2e^−^ oxygen reduction reaction (ORR) at the cathode [12]. For this reason, the AO system coupling with the electro-generation of H_2_O_2_ is called the AO-H_2_O_2_ process. H_2_O_2_, as a typical weak oxidant (E^0^ = 1.76 V/SHE), would enhance the oxidation power of the AO process by activating H_2_O_2_ to HO_2_**·**, as described in Equation (5) [13]. Furthermore, the AO-H_2_O_2_ process could be upgraded to the electro-Fenton (EF) process by the addition of metallic ions [14]. Compared to the EF system, fewer works about the alternative activation mechanism in relation to the SO_4_**·**^−^ and OH**·** originated from S_2_O_8_^2−^ (PS) and H_2_O_2_ have been done.

H_2_O → A-OH**·** + H^+^+e^−^ (1)

A-OH**·** → A=O + H^+^ + e^−^ (2)

A=O + R → R=O + A (3)

O_2_ + 2H^+^ + 2e^−^→H_2_O_2_ (4)

A + H_2_O_2_ → A-HO_2_**·** + H^+^ + e^−^ (5)

In fact, SO_4_**·**^−^ oxidation (E^0^ = 2.44 V/SHE) is considered a burgeoning AOPs with strong oxidation power, slightly milder than OH**·** oxidation (E^0^ = 2.80 V/SHE) [15]. During the AO/PS and AO-H_2_O_2_/PS processes, SO_4_**·**^−^ can be formed through several representative activation methods, including thermal activation via Equation (6) [16], reduction at cathode via Equation (7) [17], reaction with H_2_O_2_ or organics (R) via Equation (8) and via Equation (9) [18,19].

S_2_O_8_^2−^ + heat → 2SO_4_**·**^−^ (6)

S_2_O_8_^2−^ + e^−^ → SO_4_**·**^−^ + SO_4_^2−^ (7)

S_2_O_8_^2−^ + H_2_O_2_ → 2SO_4_**·**^−^ +2OH**·** (8)

S_2_O_8_^2−^ + R → 2SO_4_**·**^−^ +R**·** (9)

Several studies have investigated the effect of PS activation in EAOPs, with dyes [20], pharmaceutical and personal care products (PPCPs) [21], and herbicides [22] as targeted contaminations. Surprisingly, little has been done to investigate the effect of PS activation on the degradation efficiency of the AO-H_2_O_2_/PS process, especially comparing the AO-H_2_O_2_/PS process with different anode materials. During the AO/PS and AO-H_2_O_2_/PS oxidation processes, the generation and yield of OH**·** largely depend on the nature of anode materials [23]. The generated OH**·** at the electrode surface via Equation (1) can activate PS via Equation (10) when BDD is used as an anode, while SO_4_^2−^ from Equation (7) can also be activated to SO_4_**·**^−^ by anodic oxidation via Equation (11). The combination of SO_4_**·**^−^ and OH**·** results in a complex reaction environment [24,25]. On the one hand, SO_4_**·**^−^ activated by H_2_O_2_ and reduction reaction at the cathode can improve the oxidation power of the AO/PS and AO-H_2_O_2_/PS processes. On the other hand, SO_4_**·**^−^ generated at the anode surface will consume OH**·** in the system and occupy active anode sites, competing with contamination oxidation.

S_2_O_8_^2−^ + A-OH**·** → A + SO_4_**·**^−^ + HSO_4_^−^ (10)

SO_4_^2−^ → SO_4_**·**^−^ + e^−^ (11)

This work aims to assess the effect of PS activation by electrogenerated H_2_O_2_ and anodic oxidation on the degradation efficiencies of the AO/PS and AO-H_2_O_2_/PS processes with two typical anodes, Pt and BDD, using tartrazine azo dye as targeted contamination. Comparative trials were made in the present study to clarify the role of electrogenerated A-OH**·**, OH**·,** and SO_4_**·**^−^ on the decolorization efficiency and mineralization of tartrazine solution. The effects of PS concentration, current density, and temperature on the decolorization performance were evaluated.

## 2. Materials and Methods

### 2.1. Materials

Tartrazine (>95%) was purchased from Macklin (China) and used without any further purification. Sodium sulfate and sodium persulfate used as supporting electrolytes were obtained from Sinopharm Chemical Reagent Beijing Co., Ltd. (Beijing, China). Sulfuric acid and sodium hydroxide used for adjustment of solution pH were purchased from Sinopharm Chemical Reagent Beijing Co., Ltd. Analytical standards methanol and tert-butanol were purchased from Aladdin (China). BDD and the platinum plate used as the anode material was supplied by NeoCoat (Switzerland) and General Research Institute for Nonferrous Metals (Beijing, China), respectively. Graphite felt (GF), supplied by Jiuhua Carbon (Xiangtan, China), was used as a cathode. Distilled water obtained from Watsons was used to prepare all solutions in this study.

### 2.2. Electrochemical Oxidation Experiments

The electrochemical oxidation of tartrazine was performed in an undivided plexiglass-made reactor with a 300 mL capacity. During the oxidation process, tartrazine solutions with different electrolytes were under vigorous magnetic stirring in a water bath kettle to keep mass transfer and temperature. The electrochemical oxidation experiments were conducted under galvanostatic mode with an ITECH IT6333A (USA) to supply power. The reactor was equipped with either a Pt plate (2.5 cm × 5 cm) or a BDD electrode (2.5 cm × 5 cm) as the anode; the immersed area of all anodes was 10 cm^2^. During the AO/PS process, the cathode was a graphite cathode with an immersed area of 10 cm^2^ (2.5 cm × 5 cm), which was substituted by larger graphite (immersed area of 36 cm^2^, 6 cm× 6 cm) fed with air at a flow rate of 1 L min^−1^ to carry out the AO-H_2_O_2_/PS trials. The distance between the active sides of the anode and cathode was kept at about 1 cm, and the initial pH value of the working solution was adjusted to 6.8 with NaOH or H_2_SO_4_. Samples, filtered through a 0.22 μm filter, were taken during the oxidation process at certain time intervals to analyze the color removal.

### 2.3. Analytic Methods

The absorbance of filtered samples with 20 times dilution was determined by a Metash UV-8000 Spectrophotometer (Shanghai, China) with the detector λ = 429 nm using a standard quartz cuvette (1 cm of the optic path). The percentage of color removal was calculated as follows:

(12) $$\mathrm{Color}\;\mathrm{Removal}=\frac{A_{0}-A}{A_{0}}\times 100$$

The electrogenerated H_2_O_2_ was determined by a standard spectrophotometric method detecting the absorbance of yellowish Ti (Ⅳ) complex using a Metash UV-8000 Spectrophotometer (Shanghai, China) with the detector λ = 408 nm.

## 3. Results and Discussion

### 3.1. Effect of PS Concentration on Color Removal

**Figure 1:**
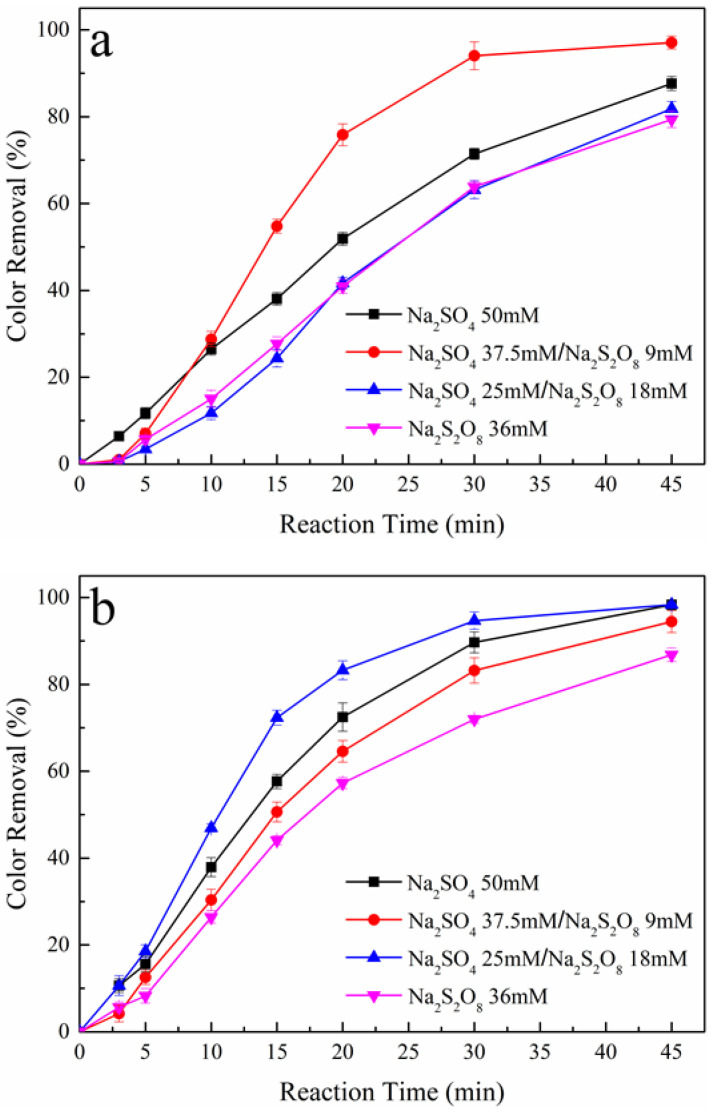

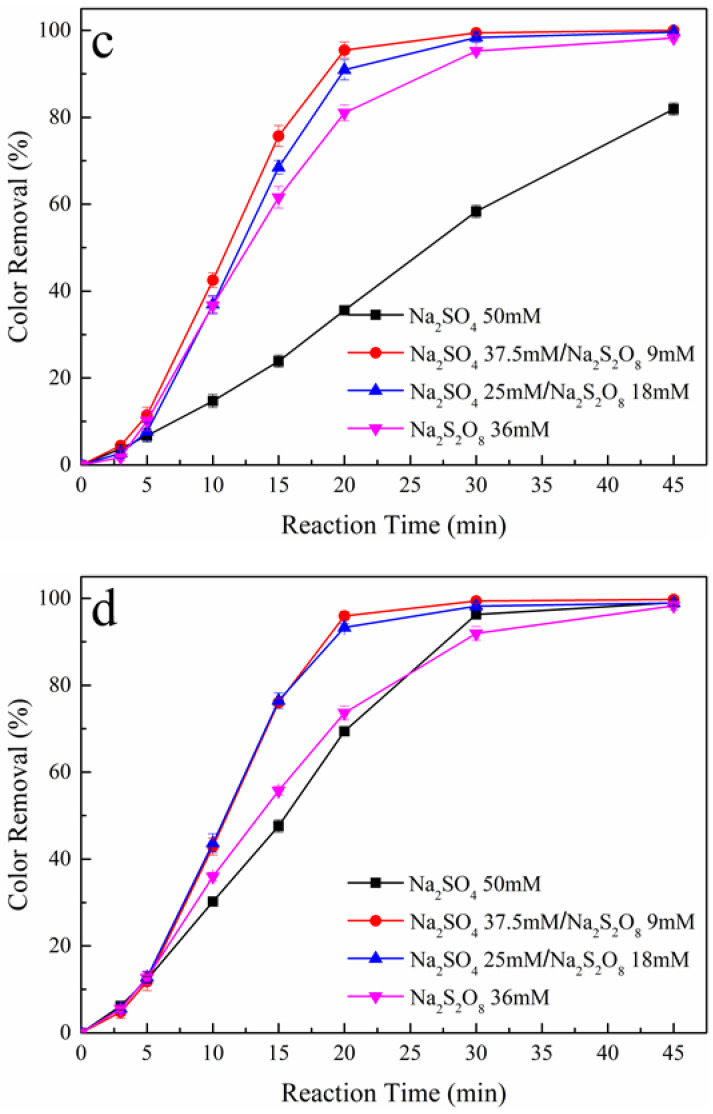
Effect of PS concentration on color removal during the AO/PS and AO-H_2_O_2_/PS processes, (**a**) Pt/PS, (**b**) BDD/PS, (**c**) Pt-H_2_O_2_/PS, and (**d**) BDD-H_2_O_2_/PS. Raw dye solution: 250 mg L^−1^ tartrazine, initial pH: 6.8, applied current density: 9 mA cm^−2^, reaction temperature: 25 °C.

The effect of PS concentration was evaluated by adding to the dye solutions four supporting electrolytes, including 50 mM Na_2_SO_4_ (0% PS), 37.5 mM Na_2_SO_4_ + 9.0 mM Na_2_S_2_O_8_ (25% PS), 25 mM Na_2_SO_4_ + 18 mM Na_2_S_2_O_8_ (50% PS) and 36 mM Na_2_S_2_O_8_ (100% PS). It is worth noting that all these four supporting electrolytes have approximately the same conductivity, between 6.9 and 7.5 mS cm^−1^ [20]. Figure 1 shows the color removal with different supporting electrolytes during the AO/PS process (Figure 1a Pt/PS, Figure 1b BDD/PS) and the AO-H_2_O_2_/PS process (Figure 1c Pt-H_2_O_2_/PS, Figure 1d BDD-H_2_O_2_/PS) at a constant current density of 9 mA cm^−2^. Table 1 shows the related degradation data during oxidation process.

The anode materials have a crucial influence on the oxidation efficiency of EAOPs [26]. To systematically research the effect of PS activation on the oxidation power of AO/PS and AO-H_2_O_2_/PS, two representative anodes, BDD and Pt, were used in this study. For the AO/PS process, it can be observed from Figure 1a,b that the color removals increased with the increase in reaction time. For reaction time of 30 min and PS concentration of 0%, 25%, 50%, and 100%, the color removals were obtained as 71%, 94%, 63%, and 64%, respectively for Pt anode, 90%, 83%, 95%, and 72%, respectively for BDD anode. It is evident that the appropriate addition of PS can increase the oxidation power of the AO/PS process, with the optimal concentration of PS being 25% for Pt and 50% for BDD. This phenomenon can be ascribed to the generated SO_4_**·**^−^ via Equations (6), (7), and (10). Nevertheless, excessive dosing of PS leads to an obvious decrease in decolorization because of the competitive effect between PS and tartrazine on the anode surface. For the Pt anode, the decolorization is a mediated electrolysis with tartrazine oxidized by A=O [27]; the generation of SO_4_**·**^−^ largely depends on reduction at the cathode via Equation (7). However, for the BDD anode, the oxidation of tartrazine relies on the electrogenerated A-OH**·** [28], and SO_4_**·**^−^ generated through Equations (7) and (10) promotes the color removal. Due to the disparate generation pathway of SO_4_**·**^−^ and differences in properties of anode materials, distance exists between the optimal concentration of the Pt/PS and BDD/PS processes.

For the AO-H_2_O_2_/PS process, at a reaction time of 20 min and PS concentration of 0%, 25%, 50%, and 100%, the color removals were obtained as 36%, 95%, 91%, and 81%, respectively for the Pt anode, 69%, 96%, 93%, and 74%, respectively for the BDD anode, as shown in Figure 1c,d. It can be observed that the additions of PS tremendously improve the oxidation power of the AO-H_2_O_2_/PS process. Furthermore, compared to the BDD-H_2_O_2_/PS process, the promotion of oxidation power by the addition of PS is more obvious in the Pt-H_2_O_2_/PS process. The more distinct promotion effects of PS in the AO-H_2_O_2_/PS process may cause by the activation reaction between PS and H_2_O_2_ as described in Equation (8), yielding greater productions of SO_4_**·**^−^ and OH**·** in the bulk of the reaction solution. Surprisingly, excessive dosing of PS still results in a decrease in decolorization in the AO-H_2_O_2_/PS process. This phenomenon confirms the governing factor of active radicals generation via Equation (8) is the concentration of H_2_O_2_. In the AO-H_2_O_2_/PS process, the accumulation of H_2_O_2_ is a relatively slow course, especially with a carbonaceous cathode without any modification [29]. In this study, almost total decolorization can be obtained at a reaction time of 45 min. The accumulated concentration of H_2_O_2_ was just 56 mg L^−1^ in 50 mM Na_2_SO_4_ solution without tartrazine at a reaction time of 45 min. For the Pt-H_2_O_2_/PS process, adding PS is an effective strategy to improve color removal since the generations of SO_4_**·**^−^ and OH**·** rely on Equation (8) to introduce large numbers of free radicals into the system. However, for the BDD-H_2_O_2_/PS process, this improvement effect is not obvious compared with the Pt-H_2_O_2_/PS process. This trend may be associated with the competitive effect between PS and tartrazine on the anode surface, as described in Equation (10). Therefore, 37.5 mM Na_2_SO_4_ + 9.0 mM Na_2_S_2_O_8_ (25% PS) and 25 mM Na_2_SO_4_ + 18 mM Na_2_S_2_O_8_ (50% PS) were chosen as the optimal supporting electrolytes to investigate the influence of applied current density for the Pt and BDD anode, respectively.

### 3.2. Effect of Applied Current Density on Color Removal

**Figure 2:**
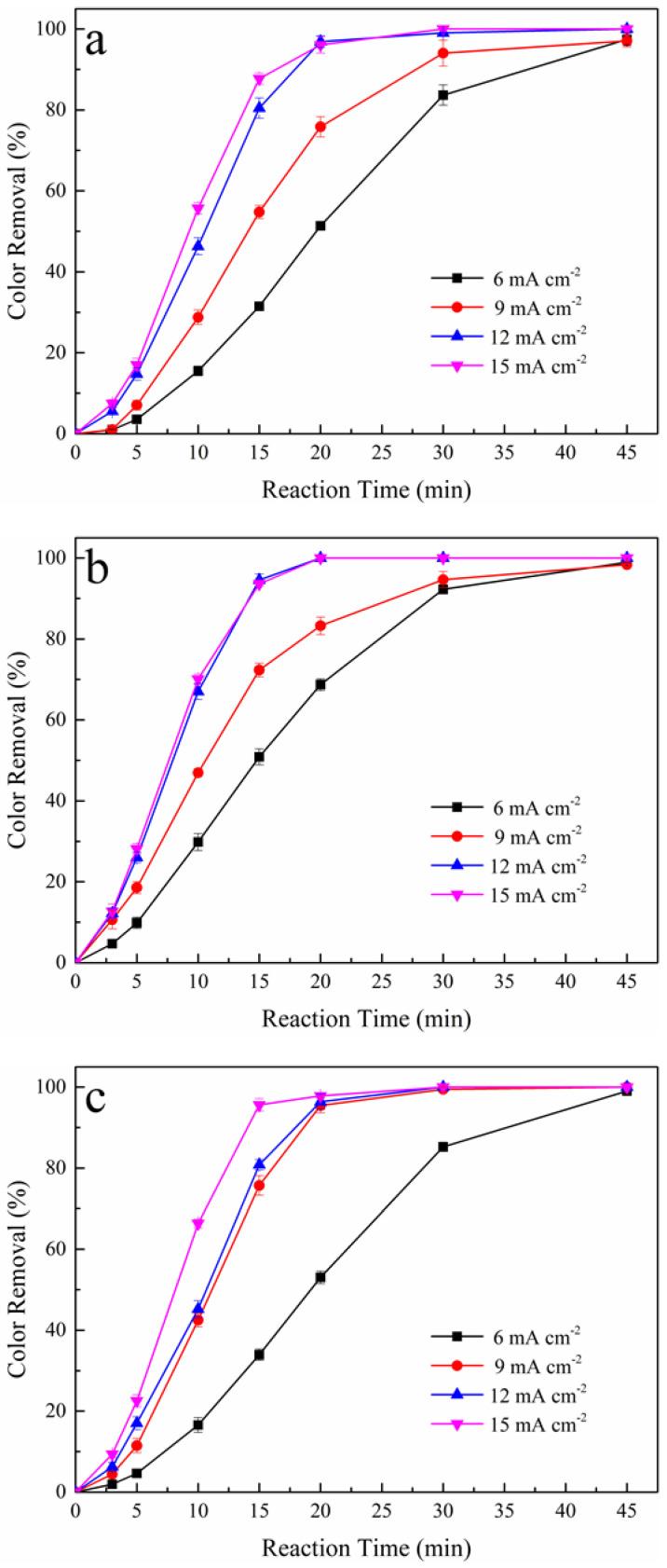

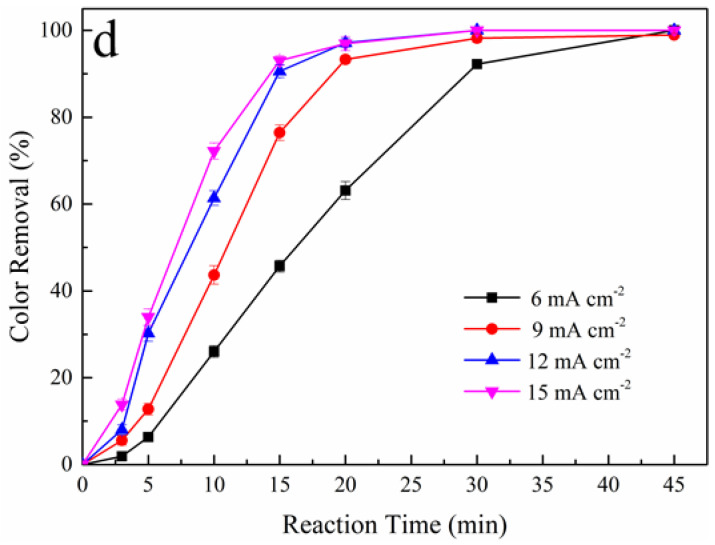
Effect of applied current density on color removal during AO/PS and AO-H_2_O_2_/PS processes, (**a**) Pt/PS, (**b**) BDD/PS, (**c**) Pt-H_2_O_2_/PS, and (**d**) BDD-H_2_O_2_/PS. Raw dye solution: 250 mg L^−1^ tartrazine, initial pH: 6.8, reaction temperature: 25 °C.

Current density, the applied current carried by per unit area of the electrode, is the most vital parameter in EAOPs [30]. In the AO/PS and AO-H_2_O_2_/PS processes, current density regulates electron transfer and the production of OH**·** at the anode and H_2_O_2_ at the cathode [31]. Figure 2 shows the effect of applied current density ranging from 6 to 15 mA cm^−2^ on the color removal of tartrazine solutions in the AO/PS process and the AO-H_2_O_2_/PS process. Table 2 shows the related degradation data during oxidation process.

For the Pt/PS process, at a reaction time of 20 min, PS concentration of 25% and applied current densities of 6, 9, 12, and 15 mA cm^−2^, the color removals were obtained as 51%, 76%, 97%, and 96%, respectively, as shown in Figure 2a. Furthermore, for the BDD/PS process, at a reaction time of 20 min, PS concentration of 50%, and applied current densities of 6, 9, 12, and 15 mA cm^−2^, the color removals were obtained as 68%, 83%, and 100%, respectively, as shown in Figure 2b. Generally, a gradual increase in decolorization can be noticed with the increase of the current density from 6 to 12 mA cm^−2^. For the Pt/PS process, this phenomenon may be caused by the progressive generation of SO_4_**·**^−^ via Equation (7) at the cathode and A=O at the anode. For the BDD/PS process, the promotion effect may be ascribed to the growing yield of A-OH**·** via Equation (1) at the anode and SO_4_**·**^−^ via Equations (7) and (10). However, the color removal of tartrazine solution remained stable with the current density increased from 12 to 15 mA cm^−2^ in the Pt/PS process and the BDD/PS process. The abovementioned tendency can be attributed to the parasitic reactions at the anode and the cathode at high applied current density [32]. With the increase of current density, the hydrogen evolution reaction at the cathode and oxygen evolution reaction at the anode surface are enhanced, competing with the production of OH**·** and SO_4_**·**^−^. The hydrogen evolution reaction at the cathode and oxygen evolution reaction at a higher current density might inhibit the oxidation of tartrazine during the AO/PS process, leading to stable color removal with a higher current density.

For the Pt-H_2_O_2_/PS process, at a reaction time of 20 min, PS concentration of 25%, and applied current densities of 6, 9, 12, and 15 mA cm^−2^, the color removals were obtained as 53%, 95%, 96%, and 97%, respectively. Figure 2c highlights a sharp increase in decolorization in the Pt-H_2_O_2_/PS process with the increase of the current density from 6 to 9 mA cm^−2^. This phenomenon may be attributed to the sharp increase in the accumulated concentration of H_2_O_2_, with the concentration of 19 mg L^−1^ in the Pt-H_2_O_2_/PS process without tartrazine at a current density of 6 mA cm^−2^ increased to 38 mg L^−1^ at a current density of 9 mA cm^−2^ at a reaction time of 45 min. However, further increase of current density to 12 mA cm^−2^ merely caused a slight increase of H_2_O_2_, with accumulated concentration increased to 44 mg L^−1^. With the increase of current density, the hydrogen evolution reaction at the cathode is promoted, inhibiting the electro-generation of H_2_O_2_ via Equation (4).

For the BDD-H_2_O_2_/PS process, at a reaction time of 20 min, a PS concentration of 50%, and applied current densities of 6, 9, 12, and 15 mA cm^−2^, the color removals were obtained as 63%, 93%, 96%, and 97%, respectively, as shown in Figure 2d. The effect of current density in the BDD-H_2_O_2_/PS process presented an analogical tendency, as shown in the Pt-H_2_O_2_/PS process. The accumulated concentration of H_2_O_2_ in the BDD-H_2_O_2_/PS process without tartrazine at 6 mA cm^−2^ is 13 mg L^−1^, increasing to 34 mg L^−1^ at a current density of 9 mA cm^−2^ at a reaction time of 45min. Further increase of current density to 12 mA cm^−2^, the accumulated concentration only increased to 40 mg L^−1^. Due to the strong oxidizing capacity of the BDD anode, electrogenerated H_2_O_2_ can be oxidized by A-OH**·** at the BDD surface via Equation (13) [33]. Hence, the concentration of H_2_O_2_ in the BDD-H_2_O_2_/PS process is lower than that in the Pt-H_2_O_2_/PS process. Based on the above discussion, applied current density has a significant influence on color removal, and the optimum value for the AO/PS and AO-H_2_O_2_/PS processes was 12 mA cm^−2^.

H_2_O_2_ + A-OH**·** → A-HO_2_**·** + H_2_O (13)

### 3.3. Effect of Reaction Temperature on Color Removal

Compared with other free radicals, PS can be activated by heat to generate SO_4_**·**^−^, a relatively low-cost and efficient activation method [34]. The effect of reaction temperature on color removal ranged from 25 to 45 °C in the AO/PS process and the AO-H_2_O_2_/PS process, considering the tolerance and practicality of the experimental device. Table 3 shows the related degradation data during oxidation process.

**Figure 3:**
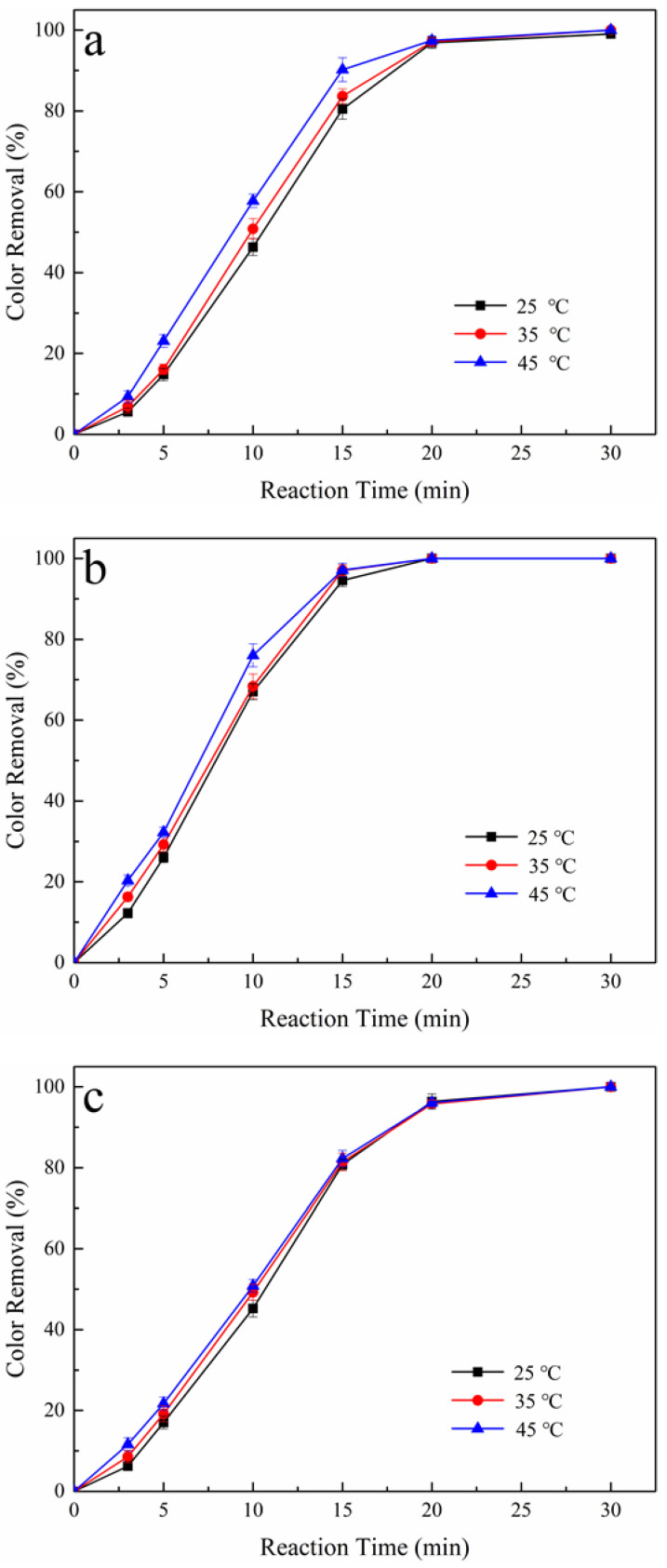

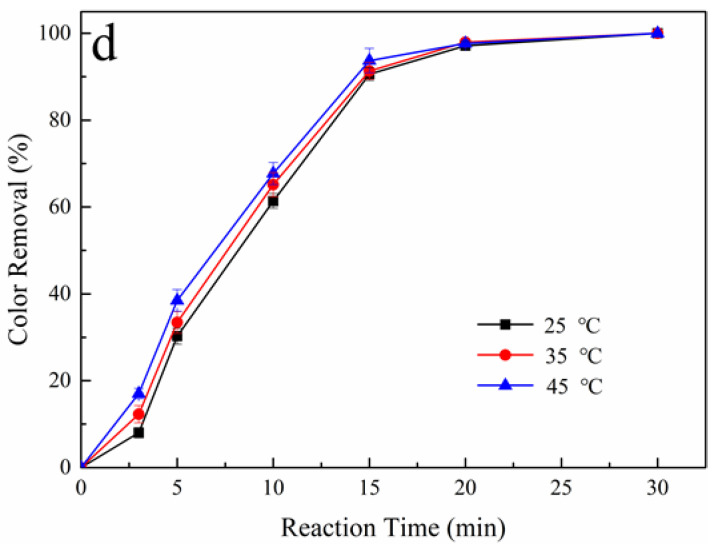
Effect of reaction temperature on color removal during AO/PS and AO-H_2_O_2_/PS processes, (**a**) Pt/PS, (**b**) BDD/PS, (**c**) Pt-H_2_O_2_/PS, and (**d**) BDD-H_2_O_2_/PS. Raw dye solution: 250 mg L^−1^ tartrazine, initial pH: 6.8, applied current density: 12 mA cm^−2^.

For the Pt/PS process, at a reaction time of 15 min, PS concentration of 25%, and reaction temperature of 25, 35, and 45 °C, the color removals were obtained as 80%, 84%, and 90%, respectively, as shown in Figure 3a. A slight increase in color removal can be attained with the increase of the reaction temperature, reflecting that the generation of SO_4_**·**^−^ activated by heat in the Pt/PS process occupied a relatively small proportion. Although higher temperatures can activate PS and enhance mass transfer during EAOPs, the critical factor affecting oxidation performance is the highly active oxidants absorbed at the anode surface [35]. A similar phenomenon was observed in the BDD/PS process, as shown in Figure 3b. At a reaction time of 15 min, PS concentration of 50%, and reaction temperatures of 25, 35, and 45 °C, the color removals were obtained as 94%, 97%, and 98%, respectively. Adding PS can increase the oxidation power of the AO/PS process at the Pt or BDD anodes. Still, the oxidation of tartrazine largely relies on the electrogenerated oxidants absorbed at the anode surface; SO4 **·**^−^ primarily comes from Equation (7) and can only promote color removal. From this discussion, one can deduce that the main source of SO_4_**·**^−^ in the AO/PS process is PS reduction at the cathode.

For the Pt-H_2_O_2_/PS process, at a reaction time of 15 min, a PS concentration of 25%, and reaction temperatures of 25, 35, and 45 °C, the color removals were obtained as 80%, 81%, and 82%, respectively. Figure 3c exhibits a nearly similar loss of color in the Pt-H_2_O_2_/PS process with the increased reaction temperature. The slight increase in color removal may be caused by the almost unchanged concentration of H_2_O_2_ accumulated in the Pt-H_2_O_2_/PS process without tartrazine. At a reaction temperature of 25 °C, the concentration of H_2_O_2_ was 44 mg L^−1^ after 45 min of electrolysis. Further increasing the temperature to 35 and 45 °C, the concentrations of H_2_O_2_ were 47 and 48 mg L^−1^, respectively. This phenomenon may be attributed to the vital factors that affect the electrogenerated efficiency of H_2_O_2_. The key factors influencing the concentration of H_2_O_2_ in the AO-H_2_O_2_ and EF processes are the applied current density and the concentration of dissolved oxygen in the solution [36]. For the BDD-H_2_O_2_/PS process, the analogical tendency, as shown in Figure 3d, may also be caused by the concentration of H_2_O_2_ accumulated in solution without tartrazine, with the accumulated concentrations of H_2_O_2_ in the BDD-H_2_O_2_/PS process at reaction temperatures of 25, 35, and 45 °C were 40, 42, and 39 mg L^−1^, respectively.

### 3.4. Oxidation Mechanism of Tartrazine in AO/PS and AO-H_2_O_2_/PS Process

The recipient standpoint of contaminations by the AO process in an aqueous solution is indirect oxidation, with the oxidation of contaminations initiated by reactive intermediates generated on the anode surface, including A-OH**·** and A=O. Furthermore, with the addition of PS, SO_4_**·**^−^ is introduced into the oxidation mechanism. To investigate the competitive generation of A-OH**·**, OH**·,** and SO_4_**·**^−^ in the AO/PS and AO-H_2_O_2_/PS processes, two well-known free radical scavengers, tert-butanol and methanol, were used. Tert-butanol was used to scavenge A-OH**·** and OH**·**, whereas methanol was used to scavenge all these radicals [20]. For this purpose, all these radicals were exhaustively scavenged; the concentrations of tert-butanol and methanol are 500 times greater than that of tartrazine.

**Figure 4:**
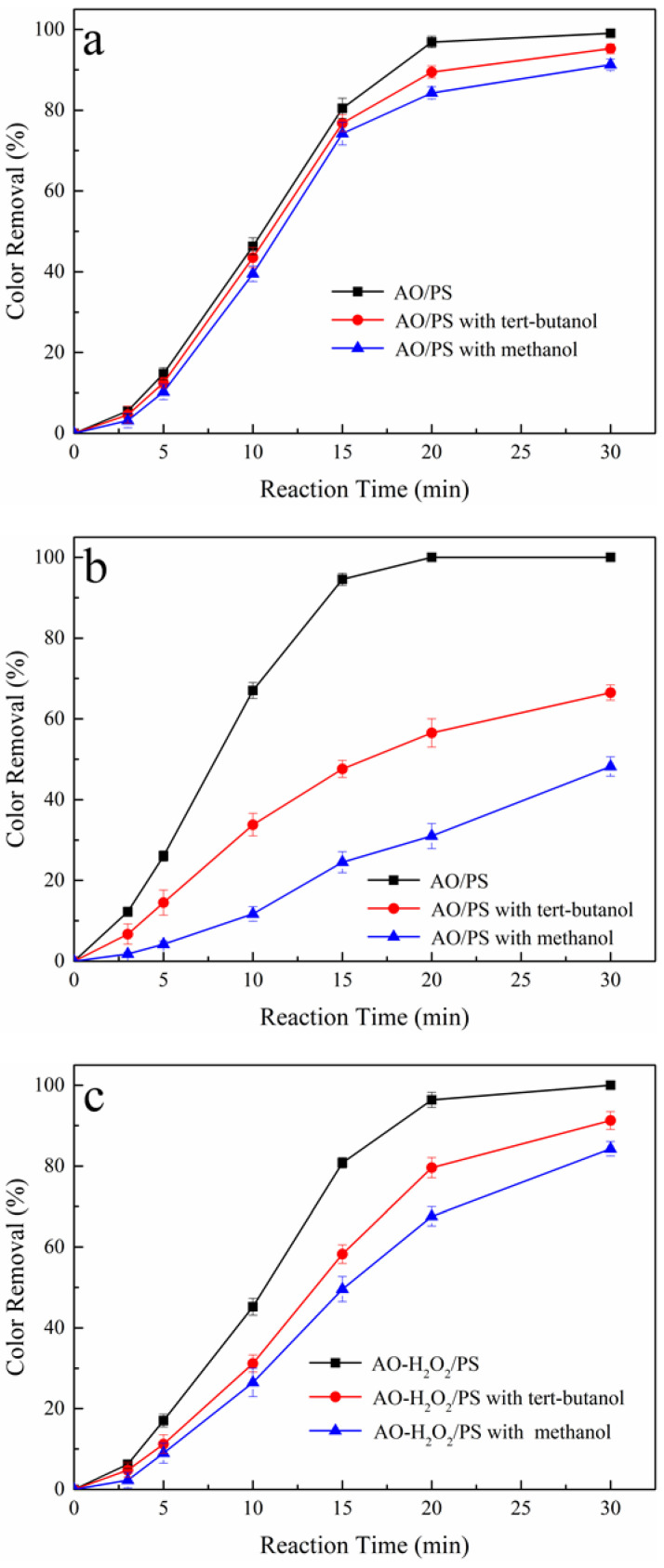

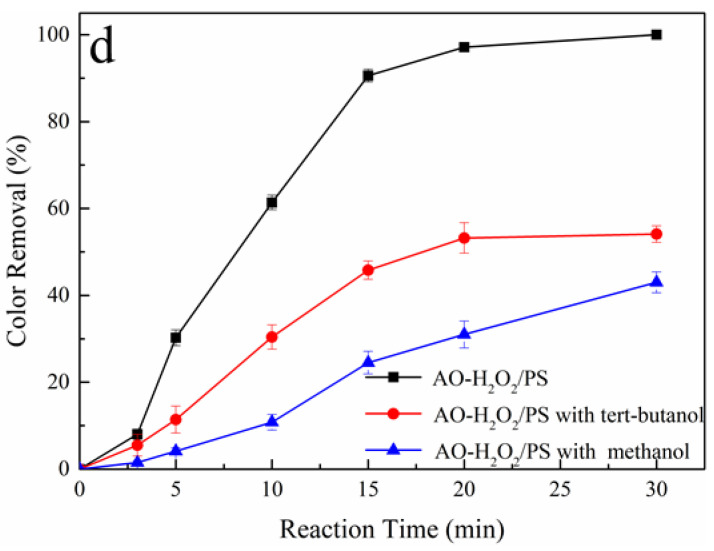
Effect of free radical scavengers on color removal during AO/PS and AO-H_2_O_2_/PS processes, (**a**) Pt/PS, (**b**) BDD/PS, (**c**) Pt-H_2_O_2_/PS, and (**d**) BDD-H_2_O_2_/PS. Raw dye solution: 250 mg L^−1^ tartrazine, initial pH: 6.8, reaction temperature: 25 °C, applied current density: 12 mA cm^−2^.

As shown in Figure 4a, the color removals were slightly affected by the addition of tert-butanol and methanol, indicating the low yields of A-OH**·**, OH**·,** and SO_4_**·**^−^ in the Pt/PS process. However, the loss of color removals in the BDD/PS process was more pronounced in the presence of tert-butanol and methanol, as shown in Figure 4b. The differences in color removals between the Pt/PS and BDD/PS processes with free radical scavengers may be ascribed to the property’s discrepancy of Pt and BDD anodes. The decolorization at the Pt anode is a mediated electrolysis by A=O and SO_4_**·**^−^ primarily comes from reduction reaction at the cathode via Equation (7). Nevertheless, the oxidation of tartrazine largely depends on the electrogenerated A-OH**·** at the BDD anode and SO_4_**·**^−^ generated through Equations (7) and (10).

For the Pt-H_2_O_2_/PS process, a large decrease in final decolorization was observed compared to the Pt/PS process, as shown in Figure 4c. The decrease in oxidation power by the addition of free radical scavengers may cause by the activation reaction between PS and H_2_O_2_ as described in Equation (8), indicating that SO_4_**·**^−^ and OH**·** in the bulk of reaction solution promoted the color removal in the Pt-H_2_O_2_/PS process. Furthermore, the loss of color removals in the BDD-H_2_O_2_/PS process was more pronounced in the presence of tert-butanol and methanol, as shown in Figure 4d. According to this phenomenon, one can conclude that the color removal in the BDD-H_2_O_2_/PS process is dominated by these free radicals, especially A-OH**·**, OH**·,** and SO_4_**·**^−^. The remaining color removal in the BDD-H_2_O_2_/PS process with radical scavengers may be caused by H_2_O_2_ and A-HO_2_**·**.

**Figure 5:**
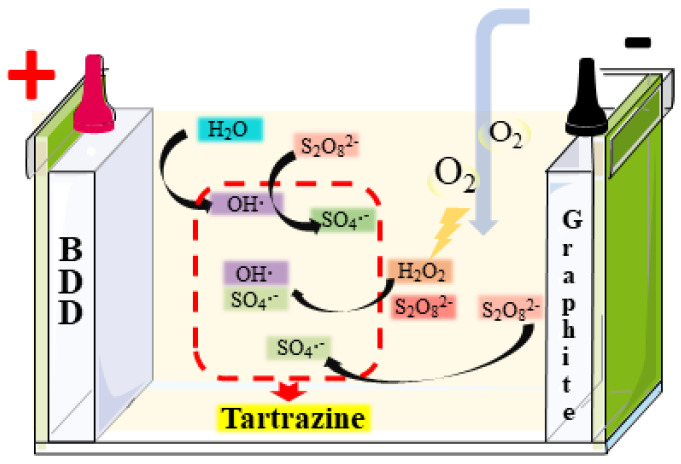
Proposed electrochemical oxidation mechanism in AO/PS and AO-H_2_O_2_/PS process.

Based on the above analysis, these oxidizing agents played an important role in the BDD/PS, Pt-H_2_O_2_/PS, and BDD-H_2_O_2_/PS processes. During the Pt/PS process, the decolorization is caused by A=O and SO_4_**·**^−^, primarily coming from the reduction reaction at the cathode via Equation (7). In the BDD/PS process, the oxidation of tartrazine largely depends on the electrogenerated A-OH**·** at the BDD anode and SO_4_**·**^−^ generated through Equations (7) and (10). However, for the AO-H_2_O_2_/PS process, adding PS is an effective strategy to improve color removal since the generations of SO_4_**·**^−^ and OH**·** rely on Equation (8) to introduce large numbers of free radicals into the system. Based on the above analysis, the promotive effect of PS at Pt and BDD anodes during the AO and AO-H_2_O_2_ processes is proposed in Figure 5.

To confirm the effect of dissolved oxygen in the AO-H_2_O_2_/PS system, N_2_ was passed into the reaction solution at a flow rate of 1 L min−1 during the Pt-H_2_O_2_/PS process and in the BDD-H_2_O_2_/PS process instead of the air. For the Pt-H_2_O_2_/PS process, at a reaction time of 15 min, PS concentration of 25%, applied current density: 12 mA cm^−2,^ and reaction temperature of 25 °C, the color removals were obtained as 75%, lower than that of the Pt/PS process. For the BDD-H_2_O_2_/PS process, the same phenomenon was noticed, as the color removals were only 79% lower than that of the BDD/PS process. N_2_ experiments proved that dissolved oxygen promoted the degradation of tartrazine in the AO-H_2_O_2_/PS system.

**Figure 6:**
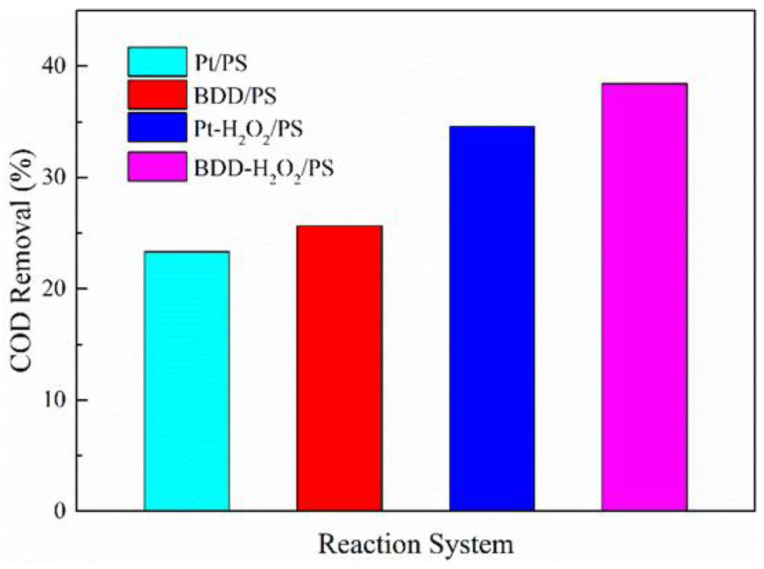
COD removal efficiency in AO system. Raw dye solution: 250 mg L^−1^ tartrazine, initial pH: 6.8, reaction temperature: 25 °C, applied current density: 12 mA cm^−2^.

Tartrazine can be completely removed in AO/PS and AO-H_2_O_2_/PS system after 45 min oxidation. However, the decoloration process of dyes by AO and related technologies is usually detected from the abatement of the COD values. To evaluate the oxidation ability of the AO/PS and AO-H_2_O_2_/PS system on the COD, the oxidation experiments in aqueous solutions containing 250 mg L^−1^ tartrazine under an applied current density of 12 mA cm^−2^ and initial pH value of 6.8 were conducted. The variations of COD values with different oxidation systems are shown in Figure 6. For the AO/PS process, at a reaction time of 45 min, the COD removals were obtained as 23% for the Pt and 26% for the BDD anode. For the AO-H_2_O_2_/PS process, at a reaction time of 45 min, the COD removals were obtained as 34% for the Pt and 38% for the BDD anode. Hence, we could deduce that tartrazine was more effectively degraded and oxidized during the AO process than oxidation products.

### 3.5. Economic Analysis

During EAOPs, it was clear that the main cost was power and electrode consumption. Considering the energy and PS consumption, the direct cost of tartrazine treatment by the BDD-H_2_O_2_/PS process was calculated. The electricity cost for treating 1 L of tartrazine solutions by the BDD-H_2_O_2_/PS process under the optimum conditions was $0.0002. The PS cost for treating 1 L of tartrazine solutions by the BDD-H_2_O_2_/PS process under the optimum conditions was $0.0051. Hence, the oxidation cost of tartrazine by BDD-H_2_O_2_/PS was $0.0053 per L. Other oxidation methods for treatment of refractory organics have been reported, such as coagulation cascaded with granular activated carbon, O_3_/H_2_O_2,_ and solar photo-Fenton, with the corresponding treatment costs of $0.0166, $0.0125, and $0.0147 [37] per L, respectively. Compared with traditional catalytic oxidation technologies, EAOPs hardly require a catalyst, and the electrode lifetime is much longer than that of a catalyst. Therefore, the cost of an electrode can be reduced by the catalyst cost and the usage of alternate forms of energy (e.g., solar energy and wind energy), and to confirm the stability of the AO/PS and AO-H_2_O_2_/PS system, 5 consecutive cycles experiments were conducted. Thanks to the stability of commercial anodes (Pt and BDD), no decreasing signs of dye degradation efficiency were observed, indicating that the AO/PS and AO-H_2_O_2_/PS systems have good stability and meet the requirement of long-term continuous operation.

## 4. Conclusions

It has been demonstrated that activating PS by electrogenerated H_2_O_2_ and anodic oxidation promotes the decolorization of tartrazine solutions at the Pt and BDD anodes. For the Pt anode, SO_4_**·**^−^ was generated from the reduction reaction at the cathode and reaction between PS and H_2_O_2_ with the optimal concentration of PS at 25%. For the BDD anode, SO_4_**·**^−^ was generated from the reduction reaction at the cathode, oxidation reaction at the anode, and reaction between PS and H_2_O_2_ with the optimal concentration of PS at 50%. Among these activation methods, the activation efficiency of PS by H_2_O_2_ is much better than other methods. The processes become more efficient based on the increase of the applied current density to 12 mA cm^−2^ due to the more production of SO_4_**·**^−^ and OH**·** under high current density. The PS activations by thermal show much the same performance with the temperature range from 25 to 45 °C. The main oxidizing agents are SO_4_**·**^−^ and OH**·** in AO/PS and AO-H_2_O_2_/PS, and the yields of these radicals in the AO/PS process are relatively low.

## Figures and Tables

**Table 1:** Effect of PS concentration on color removal during AO/PS and AO-H2O2/PS processes.

Oxidation System	0% PS	25% PS	50% PS	100% PS
	Color Removal (%) *			
Pt/PS	71	94	63	64
BDD/PS	90	83	95	72
Pt-H_2_O_2_/PS	36	95	91	81
BDD-H_2_O_2_/PS	69	96	93	74

* For Pt/PS and BDD/PS, data of 30 min was selected. For Pt t-H_2_O_2_/PS and BDD t-H_2_O_2_/PS, data of 20 min was selected.

**Table 2:** Effect of applied current density on color removal during AO/PS and AO-H_2_O_2_/PS processes.

Oxidation System	6 mA cm^−2^	9 mA cm^−2^	12 mA cm^−2^	15 mA cm^−2^
	Color Removal (%) *			
Pt/PS	51	76	97	96
BDD/PS	68	83	100	100
Pt-H_2_O_2_/PS	53	95	96	97
BDD-H_2_O_2_/PS	63	93	96	97

* For Pt/PS and BDD/PS, data of 20 min was selected. For Pt t-H_2_O_2_/PS and BDD t-H_2_O_2_/PS, data of 20 min was selected.

**Table 3:** Effect of reaction temperature on color removal during AO/PS and AO-H_2_O_2_/PS processes.

Oxidation System	45 °C	45 °C	45 °C
	Color Removal (%) *		
Pt/PS	80	84	90
BDD/PS	94	97	98
Pt-H_2_O_2_/PS	80	81	82
BDD-H_2_O_2_/PS	90	91	93

* For Pt/PS and BDD/PS, data of 15 min was selected. For Pt t-H_2_O_2_/PS and BDD t-H_2_O_2_/PS, data of 15 min was selected.
